# Magnetic Elements for Neuromorphic Computing

**DOI:** 10.3390/molecules25112550

**Published:** 2020-05-30

**Authors:** Tomasz Blachowicz, Andrea Ehrmann

**Affiliations:** 1 Institute of Physics–Center for Science and Education, Silesian University of Technology, 44-100 Gliwice, Poland; tomasz.blachowicz@polsl.pl; 2 Faculty of Engineering and Mathematics, Bielefeld University of Applied Sciences, 33619 Bielefeld, Germany

**Keywords:** neuromorphic computing, adaptive computing, cognitive computing, magnetism, micromagnetic simulations, magnetic nanoparticles, neural network

## Abstract

Neuromorphic computing is assumed to be significantly more energy efficient than, and at the same time expected to outperform, conventional computers in several applications, such as data classification, since it overcomes the so-called von Neumann bottleneck. Artificial synapses and neurons can be implemented into conventional hardware using new software, but also be created by diverse spintronic devices and other elements to completely avoid the disadvantages of recent hardware architecture. Here, we report on diverse approaches to implement neuromorphic functionalities in novel hardware using magnetic elements, published during the last years. Magnetic elements play an important role in neuromorphic computing. While other approaches, such as optical and conductive elements, are also under investigation in many groups, magnetic nanostructures and generally magnetic materials offer large advantages, especially in terms of data storage, but they can also unambiguously be used for data transport, e.g., by propagation of skyrmions or domain walls. This review underlines the possible applications of magnetic materials and nanostructures in neuromorphic systems.

## 1. Introduction

Conventional computers have a processing unit and a memory, used to process and to store data, respectively. Other common components are input and output mechanisms, a control unit, and a nonvolatile memory. According to the so-called von Neumann architecture, processing and storage of data are separated [1]. This leads to the “von Neumann bottleneck”, meaning that data transport is nowadays slower than data processing and storage [2]. While separate buses for data and processing instructions or parallel computing might partly solve this problem, Backus suggested in 1978 to use an alternative program architecture in combination with new rules for state transition [3]. For this approach, however, new hardware would be supportive.

One possible solution is based on the idea of neuromorphic computing, also called cognitive or adaptive computing. Brain-inspired computers could work like neuronal networks, be more energy-efficient [4], and could learn and solve special mathematical problems faster than recent computers [5]. Especially for machine vision, machine hearing, etc., neural network computations for autonomous robots, etc., this approach is often discussed [6].

Besides a few large projects, such as the Human Brain Project [7], and some commercially available bio-inspired chips, such as the TrueNorth chip [8], there are several research groups working on new hardware to enable neuromorphic computing. Diverse approaches were made to create artificial neurons (i.e., computing elements) and synapses (i.e., memory elements) as the basic modules of cognitive hardware [9,10].

Here, we concentrated on magnetic materials and structures for neuromorphic hardware, giving an overview of the most recent developments and ideas.

## 2. Magnetic Tunnel Junctions–Domain Wall Propagation and Different Switching Mechanisms

One of the ideas for data storage devices is based on domain wall motion, driven, e.g., by spin orbit torque in ferromagnet/heavy metal heterostructures [11,12]. This principle can also be used in neuromorphic computing.

**Figure 1:**
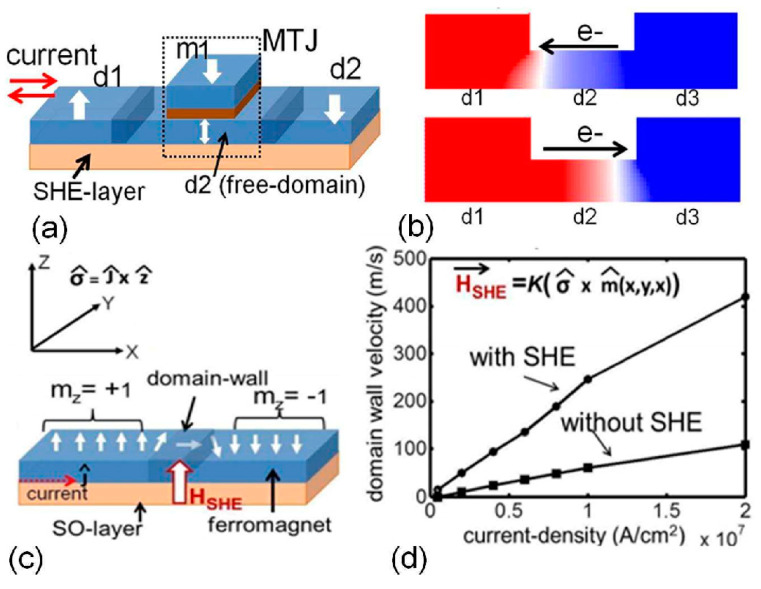
(**a**) Spin neuron with three terminals based on the domain wall motion depicted in (**b**), (**c**) contribution from the spin Hall effect (SHE) to speed up domain wall motion, (**d**) resulting domain wall motion as a function of current density. Reprinted with permission from [14]. Copyright (2018) by AIP Publishing.

Sengupta et al. suggested an all-spin artificial neural network in which domain wall propagation through ferromagnets was used to emulate neural and synaptic functionalities [13]. While common devices (Figure 1) are switched between two states [14], they suggested a multilayer consisting of a ferromagnet with a domain wall and a heavy metal layer. The latter is necessary to create a spin-orbit torque by applying a charge current, which can control the magnetic domain wall in the ferromagnet [15,16,17]. This system has the advantage of a linear, instead of a step transfer, function, which could be used for complex calculations and especially in neuromorphic computing, where such analogue behavior is often advantageous. Combining it with a pinned ferromagnetic layer on top of the free ferromagnetic layer with the domain wall, a magnetic tunnel junction (MTJ) is formed in which both ferromagnets are separated by a tunnel barrier, typically prepared from MgO or other metal oxides.

It should be mentioned that switching a layer of a magnetic tunnel junction can be done, in general, due to field-induced or current-induced effects, eventually supported by temperature (thermally assisted switching), spin transfer torque, or by the aforementioned spin-orbit torque mechanism.

Using such MTJs, Sengupta et al. proposed a device in which writing is performed by a current flow through the heavy metal underlayer, while the read current flows through the MTJ structure, perpendicular to its tunnel barrier, and is used to measure the conductance which changes typically by several 100% in such a tunnel magnetoresistance (TMR) system [18]. In this way, the domain wall position represents the synapse functionality, i.e., the memory—by changing the domain wall position, the “synaptic weight” is modified. This means that the synapse is not switched between two states, as in common computers, but has a nonstep transfer function, enabling storage of several different states. It can also be interpreted as a synapse necessitating more than one current pulse to switch between two states, as is common in the human brain. Computation, on the other hand, is performed by “writing”, i.e., shifting the domain wall position, followed by reading to enable a feedback loop. The validity of this approach was underlined by micromagnetic simulations of the domain wall motion [13].

To develop this model further, spiking neurons can be included. The idea of spiking neurons was implemented in the third generation of neural network models, while the first generation was characterized by giving only digital inputs and outputs, and the second generation had a continuous set of output values, corresponding to weighted sums of inputs, as described above and as well-known from analog computations. In the third generation, spiking neurons are used as computational units, modelling real biological neuron response functions [19].

These spikes enable single-action potentials instead of the aforementioned analog computations, applying some additional conditions. Most importantly, analog variables can now be defined by time differences between the signals instead of by adding signal heights. Technically, a threshold function can be defined, similar to biological neurons, making firing of the spiking neuron more probable when a certain time after the last firing is elapsed and nearly impossible directly afterwards [19]. Networks built of such spiking neurons were introduced in the middle of the 1990s [20,21]. Typical functionalities of such spiking neurons are integration (i.e., series of input spikes are added), firing (i.e., creating an output spike when a threshold level is reached), and ideally also leaking (i.e., reduction of the accumulated signal with time if no new input is provided).

**Figure 2:**
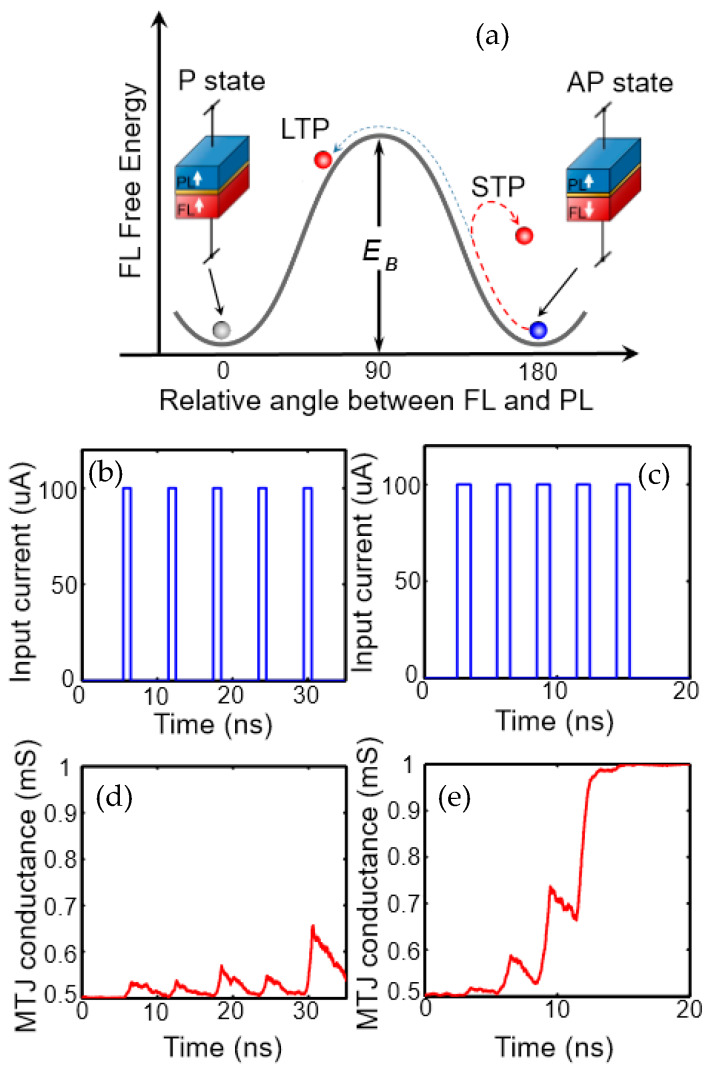
(**a**) Synaptic “learning” and “forgetting” with a mono-domain MTJ: The input stimuli frequency must be high enough to enable crossing the energy barrier separating to two stable states (STP and LTP, indicating short- and long-term plasticity). For (**b**) low input stimuli frequencies, (**d**) switching is not possible, while for (**c**) higher input frequencies, (**e**) switching is enables. Reprinted with permission from [22]. Copyright (2018) by AIP Publishing.

Using lateral spin valves, again based on the spin-orbit torque, Sengupta and Roy developed a model in which the MTJ was located at an edge of the free layer. The input spikes were given as current pulses through the heavy metal layer at different time steps, with each input spike moving the domain proportionally to the current value. When the domain wall reached the other edge of the free layer, an output spike was triggered. Interestingly, due to the pinned layer, the domain wall leaks back to its original position when the time distances between input spikes are too large, by this “leaking” even mimicking “forgetting” and not only “learning”, as depicted in Figure 2 [22]. Other papers of this group suggest further devices in a full device-circuit-system perspective, using different bio-fidelity from first generation neurons to stochastic spiking neurons [23,24].

Most recently, the domain wall synapse was investigated in detail by micromagnetic simulations using MuMax3 [25] and corresponding analytical calculations [26]. Using Pt as the heavy metal and CoFe as ferromagnet, a not fully linear dependence of the domain wall velocity on the current density was found. Using a write current pulse train, the possibility to move the domain wall in both directions could be shown. For such artificial neurons, analytical calculations and circuit simulations of a transistor-based neuron circuit were compared and found to be nearly identical. A feedback circuit evaluating the change in the synaptic weight of each node of a neural network, based on analog electronics, was also developed and calculated. The calculations showed that on-chip learning by standard neural network algorithms was possible [26].

Brigner et al. developed another system to enable the functionality of leaking without external circuitry, i.e., without an external magnetic field [27]. By using a ferromagnet with a gradient magneto-crystalline anisotropy, as it can be realized by a thickness or composition gradient [28,29], they showed the leaking functionality, i.e., the propagation of the domain wall into the original position between the spikes.

Another approach of a leaky-integrate-fire neuron was suggested by Agrawal and Roy, based on the automation of magnetic domain walls [30]. This means that the shape anisotropy in nano-magnets with high aspect ratio can support automatic movement of domain walls, even if no external magnetic field is applied. In this way, leaky-integrate-fire neurons can be approximated, enabling creating a compact and low-energy magnetic neuron, which can be cascaded up to synapse arrays. A behavioral model of such a system was shown to enable handwriting pattern recognition.

Zhang et al. went one step further. Simulating a parallel architecture based on a dense array of domain wall-based spintronics devices, they tested a selected sparse coding algorithm in terms of speed and energy dissipation. While the speed could be more than three orders of magnitude higher on this specialized parallel architecture, as compared to pure software implementation, the energy dissipation was even calculated to be eight orders of magnitude lower than for the case of software implementation. Another artificial neural circuit with a multistep transfer function, based on the same domain wall-based spintronics devices, showed good learning functionality with a good recognition error rate [31].

Besides the usually described functionalities of artificial neurons to simulate leaking, integrating, and firing, Hassan et al. investigated a possibility to add lateral inhibition without the usually applied external circuits. Simulating domain wall magnetic tunnel junction devices, they demonstrated lateral inhibition between two neighboring artificial neurons. With a trained crossbar structure consisting of 10 such neurons, they found a high signal identification rate for handwritten digits [32].

While these examples underline the possibilities of using domain wall propagation in magnetic tunnel junctions in neuromorphic computing, the next section concentrates on a different magnetic structure.

## 3. Skyrmions

Skyrmions are topologically stable solitons, firstly described by Tony Skyrme in correlation with the atomic nucleus [33]. Magnetic skyrmions were found much later [34,35] and are under examination by diverse research groups since.

**Figure 3:**
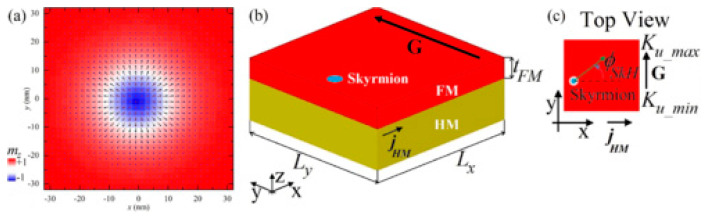
(**a**) Micromagnetic simulation of a symmetric Néel skyrmion with negative polarity; (**b**) ferromagnet/heavy metal bilayer with skyrmion, current in the heavy metal *j_HM_* and anisotropy gradient G; (**c**) top view on the ferromagnetic layer with the skyrmion Hall angle and the aforementioned driving parameters. Reprinted with permission from [40]. Copyright (2018) by the American Physical Society.

Magnetic skyrmions look, at first glance, like magnetic vortex cores, with the magnetization in their core pointing out of plane, i.e., “up” or “down” in a thin magnetic film (Figure 3). The outer edge of the skyrmion consists of spins pointing in the opposite direction. Between middle and outer edge, the magnetic moments are usually tilted along a circle around the middle or canted more and more to the inside or outside, respectively, in this way minimizing the energy of the spins between the extremal positions.

Such magnetic skyrmions can also be moved [36,37] and thus be used for data storage and processing [38]. Typical methods to drive skyrmions along a magnetic structure include a magnetic field gradient [39], an anisotropy gradient combined with the spin Hall effect (Figure 3) [40], oscillating magnetic field gradients [41], or a current [42].

Transferring these concepts into neuromorphic computing, Saxena et al. used a spin-orbit torque-driven skyrmion device as an artificial synapse in a neural network. Including material defects, they compared this skyrmion-based synapse with a domain wall-based one, as they were described above. In a simulation of a standard digit recognition problem, the skyrmion-based artificial neural network necessitated two orders or magnitude less energy than the domain wall-based one [43].

Current-driven skyrmions were used in wedge-shaped nanotracks to simulate neurons. The dynamic behavior of the skyrmion-based neurons is controlled by current pulses and repulsive forces from the edges of the nanotrack and similar to a biological neuron, i.e., includes leakage, integration, and firing, as desired. This makes such an artificial neuron a possible part of low-energy, high-density neuromorphic computing devices [44].

Design ideas for skyrmion-based devices and propagation paths were suggested by Chen et al. [45]. Using device-do-system simulations, they showed a significantly reduced energy consumption of such skyrmion-based systems, as compared to conventional computing.

**Figure 4:**
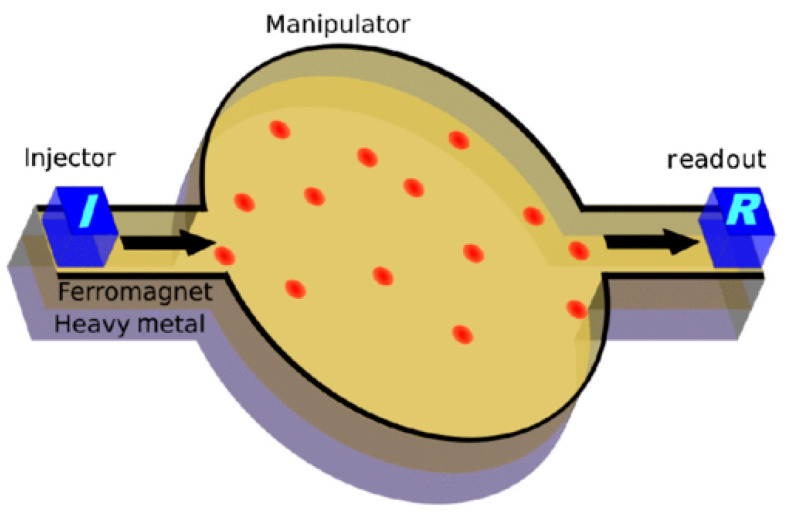
Generic skyrmion device consisting of a thin film ferromagnet/heavy metal bilayer, showing the manipulation of skyrmion between injection and readout. Reprinted with permission from [46]. Copyright (2018) by the American Physical Society.

A completely different path was proposed by Pinna et al. They prepared a skyrmion gas, i.e., a diluted irregular skyrmion lattice, and used it to copy a random signal based on skyrmion-skyrmion as well as skyrmion-edge interactions (Figure 4). This small device worked similarly to an integrate-and-fire neuron and could thus be regarded as a neuromorphic computing device [46].

**Figure 5:**
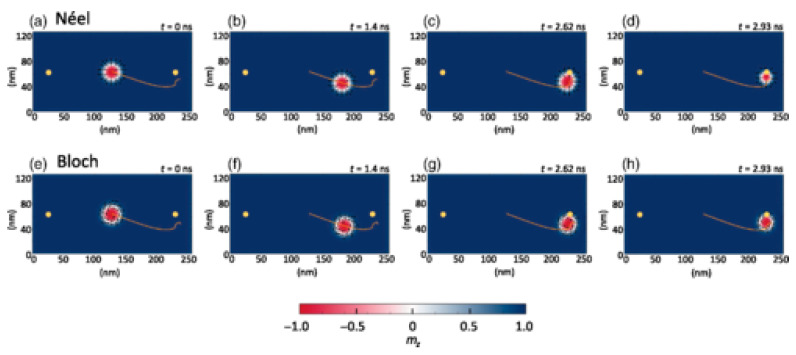
Time-dependent position of (**a**–**d**) Néel, (**e**–**h**) Bloch Skyrmion due to a fixed voltage without local pinning. Reprinted with permission from [47]. Copyright (2018) by the American Physical Society.

Skyrmions were also combined with the idea of reservoir computing, i.e., with the concept of complex artificial neural networks, modeled by recursively connected dynamical systems from self-assembled memristive devices. In such random networks, computation is encoded in the collective dynamic response of the system on an input signal. Prychynenko et al. designed a skyrmion network in a magnetic film and simulated the influence of the anisotropic magneto-resistive effect with or without (Figure 5) additional local pinning on the skyrmions in this system [47].

Li et al. suggested using a cluster of skyrmions as the base of an artificial synapse, allowing for moving skyrmions onto the device or away from it to modify the synaptic weight [48]. A full skyrmion-based artificial spiking neuron device was also proposed, realizing the leaky-integrate-fire functionality by current-driven skyrmion dynamics on a racetrack on which the moving distance defines the threshold value [49].

While the previous two sections concentrated on special magnetic structures, i.e., domain walls and skyrmions, in complex devices, the next one gives an overview of basic research on magnetic nanowires and other magnetic nanostructures which may also be used for neuromorphic computing.

## 4. Magnetic Nanowires and Other Magnetic Nanostructures

Moving magnetic domain walls is not only possible in one of the layers in a magnetic tunnel junction, but also in magnetic nanowires, nanofibers, and other nanostructured magnetic objects. Depending on the interplay between magneto-crystalline and shape anisotropy, sometimes combined with an additional magneto-elastic anisotropy or exchange anisotropy, the path of such a propagating domain wall is defined. Besides, nucleation of one or more domain walls as distinct positions or at different times can be triggered. One typical magnetic element which is often investigated by diverse research groups is the so-called Racetrack memory [50,51,52], which is planned to be used for data storage.

**Figure 6:**
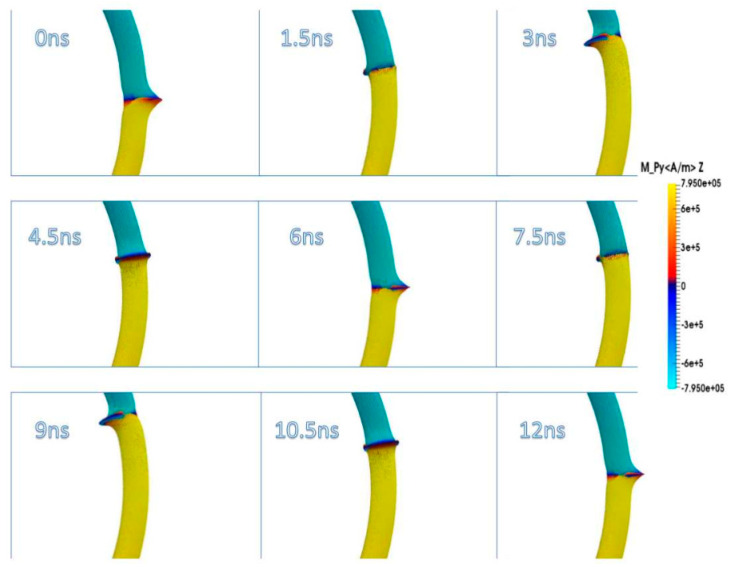
Time-dependent domain wall oscillations in a curved nanowire. Reprinted with permission from [57]. Copyright (2017) by the American Physical Society.

Due to their analog operation mode, such Racetrack memories or similar concepts can be used in neuromorphic computing for data storage and transport. Here, however, it must be taken into account that not only the cross-section, but also a bending radius of magnetic nanowires, significantly influence magnetization reversal and domain wall nucleation [53,54,55] as well as domain wall propagation (Figure 6) [56,57]. Even more challenging is the formation of logic circuits purely from magnetic nanowires [58,59,60].

**Figure 7:**
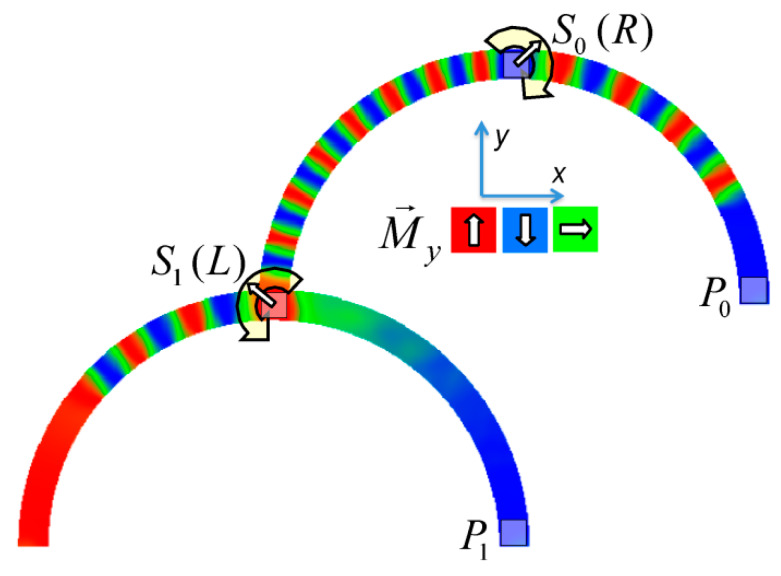
Example of double-curve system with two inputs S_i_ and two outputs P_i_.

Figure 7 depicts an example of neuro-inspired signal process by a double-curve system, simulated with the Landau-Lifshitzs-Gilbert (LLG) micromagnetic solver Magpar [61] for a permalloy sample of rectangular cross-section 10 nm × 60 nm and fiber length 1570 nm. At the positions S0 and S1, two external rotating magnetic fields are applied (1 T, 0.5 GHz). The dimensions of the local-field regions are 50 nm × 50 nm × 10 nm, identical to the sensing regions at P_0_ and P_1_. The external fields rotate in the x-y plane, either clockwise (R) or counterclockwise (L). Thus, four input combinations of first and second input are possible: LL, LR, RL, and RR, resulting in different output signals, P_0_ and P_1_. Figure 7 depicts the case of RL.

To evaluate such a system, the time-dependent output must be evaluated. Instead of showing the corresponding time-dependent magnetization curves, here we depict more intuitive snapshots of the magnetization after 10 ns and 40 ns, respectively. Table 1 shows the results of all four possible input combinations after the mentioned time spans.

Firstly, it was visible that for the cases LL and RL the lower output P_1_ did not get a signal. Rotating the lower external magnetic field S_1_ counterclockwise apparently blocked signal transport into the lower right arm of the system. Correspondingly, signal transport into the lower left arm of the system was blocked by the second signal rotating clockwise (LR and RR). In addition, there was also an influence of the first signal S_0_, as can be seen by comparing, e.g., the upper left images, depicting 10 ns after starting with LL or RL signals. This influence, however, was reduced as compared to the impact of the signal S_1_, showing that this system could not be described by a simple logic table, but rather by fuzzy logic.

## 5. Memristors and Other Nonmagnetic Neuromorphic Computing Elements

To complete this overview, a short overview of important nonmagnetic neuromorphic elements is given in this section.

The word “memristor” is composed of “memory” and “resistor” and describes, correspondingly, a storage device changing its resistance in dependence on its history, which can be defined as memory. After being described in 1971 [62], it was found only in 2007 [63]. Opposite to the above-described MTJ with a movable domain wall, the memory of the memristor works chemically, but is also reversible [64]. Its advantage is the significantly reduced energy consumption, as compared to recent DRAMs (dynamic random access memories) [65]. In addition, this “analog” behavior makes the memristor suitable for neuromorphic computing applications. A large number of publications describes the possible use of memristors as artificial synapses [66,67,68,69,70] or even neurons [71,72,73].

Alternatively, phase-change materials can be used as synapses [74,75,76].

Finally, photonic materials were also suggested as synapses [77,78,79] or neurons [78,80,81].

## 6. Conclusions

This review gave a short overview of the most recent research and developments in magnetic devices and structures for neuromorphic computing. For a review of literature from a longer time span and with a focus on magnetic tunnel junctions, upscaling problems correlated with them as well as special computing tasks for which MTJs are especially suited, the reader may be referred to [82]. While in most cases combined with nonmagnetic materials, typical magnetic structures like domain walls or skyrmions, as described in this paper, form a broad base for neuromorphic hardware elements with significantly different properties, which are important for their possible applications, as Table 2 shows.

The large interest of many research groups in this topic reveals the opportunities and challenges connected with implementing magnetic devices in more and more sophisticated real neuromorphic computers. We hope that our paper will serve as an inspiration for future research on this emerging topic.

## Figures and Tables

**Table 1:** Results of different rotational orientations at both inputs after 10 ns and 40 ns, respectively.

t (ns)	LL	RL	LR	RR
10	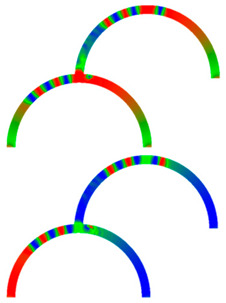	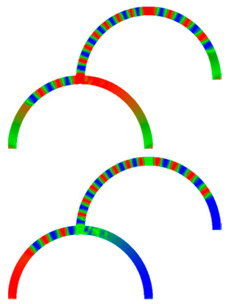	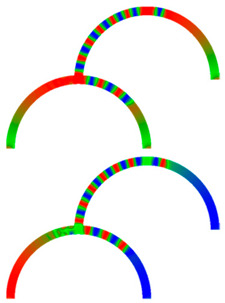	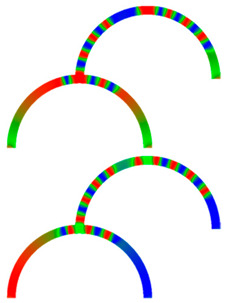
*M_x_*				
*M_y_*				
40	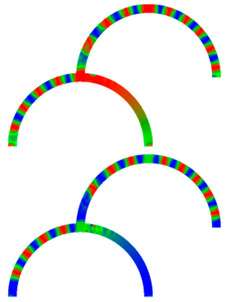	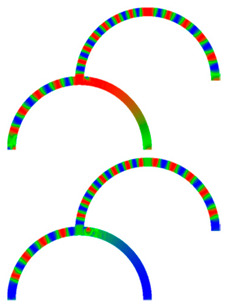	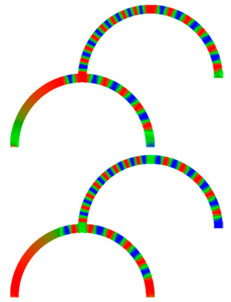	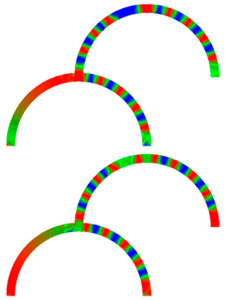
*M_x_*				
*M_y_*				

**Table 2:** Comparison of chosen magnetic elements and memristors for neuromorphic computing.

Properties	MTJ–Domain Wall Based	MTJ–Spiking Neurons	Skyrmions	Nanowires	Memristors
Endurance	High [83]	High [84]	High [85]	High [86]	High [87]
Programming accuracy	High [88]	Low, algorithmic scaling is necessary [88]	Can be high [89]	High [86]	Can be sufficiently controlled [90]
Power consumption	Low [13,83,91]	Very low (~100 nW) possible [83]	Low [92]	Low [83]	Low [93]
Speed	High (few ns) [13,83]	High (~100 ns) [88]	High [92]	Low (e.g., <100 kHz) [93]	High (~10 ns) [90]
Area consumption	Relatively high [13]	High due to high synaptic density [88]	Low [92]	Low, depends on technique [94,95]	Low [87]
Retention	<10 years [91] or more with sophisticated concepts [96]	~10 years with a sophisticated concept [96]	High [85]	~10 years [86]	Enabling short- and long-term memory [97]
Scalability	Possible [13]	Possible, but challenging [88]	Improvable based on recent findings [85]	Possible [98]	Good [87,92]
CMOS process integration	Possible [91]	Not yet possible [88]	Not possible/planned [45]	Not possible/planned [98]	Possible [93]
